# Bacterial Dysbiosis and Translocation in Psoriasis Vulgaris

**DOI:** 10.3389/fcimb.2019.00007

**Published:** 2019-02-04

**Authors:** Maria J. E. Visser, Douglas B. Kell, Etheresia Pretorius

**Affiliations:** ^1^Department of Physiological Sciences, Stellenbosch University, Stellenbosch, South Africa; ^2^School of Chemistry, The University of Manchester, Manchester, United Kingdom; ^3^The Manchester Institute of Biotechnology, The University of Manchester, Manchester, United Kingdom

**Keywords:** psoriasis, inflammation, bacteria, dysbiosis, gut microbiome, skin microbiome, bacterial translocation

## Abstract

Psoriasis vulgaris is a chronic inflammatory skin condition, associated with both a physical and a psychological burden. Our understanding of the etiology of this disease remains incomplete. Conventionally, psoriasis has been viewed as a condition that manifests solely in the skin. However, the systemic inflammatory nature of this disease has been confirmed by the presence of a wide array of dysregulated cytokines and inflammatory markers in the serum of these patients. Both dysregulated gut and skin microbiomes have been found in association with psoriasis. An evident association also exists between inflammatory bowel disease and this condition. Regarding the skin microbiome, changes have been observed in the relative abundance of Firmicutes, Actinobacteria, and Proteobacteria. Additionally, *Staphylococcus* and *Streptococcus* spp. were detected more frequently in lesional skin. Alterations in the gut microbiome have been characterized by a decrease in the Bacteroidetes phylum and an increase in the *Faecalibacterium* genus. We suggest that dysbiosis of the skin and gut microbiota may contribute to psoriasis, by promoting the translocation of microbes from these sites into the bloodstream. Consistent with the Iron Dysregulation and Dormant Microbes hypothesis, these microorganisms are in a physiologically dormant state, but may be awakened periodically and shed their cell wall components, such as lipopolysaccharide and lipoteichoic acid. Both of these inflammagens may contribute significantly to maintaining a chronic inflammatory state in the host, such as is seen in individuals diagnosed with psoriasis.

## Introduction

**Figure 1:**
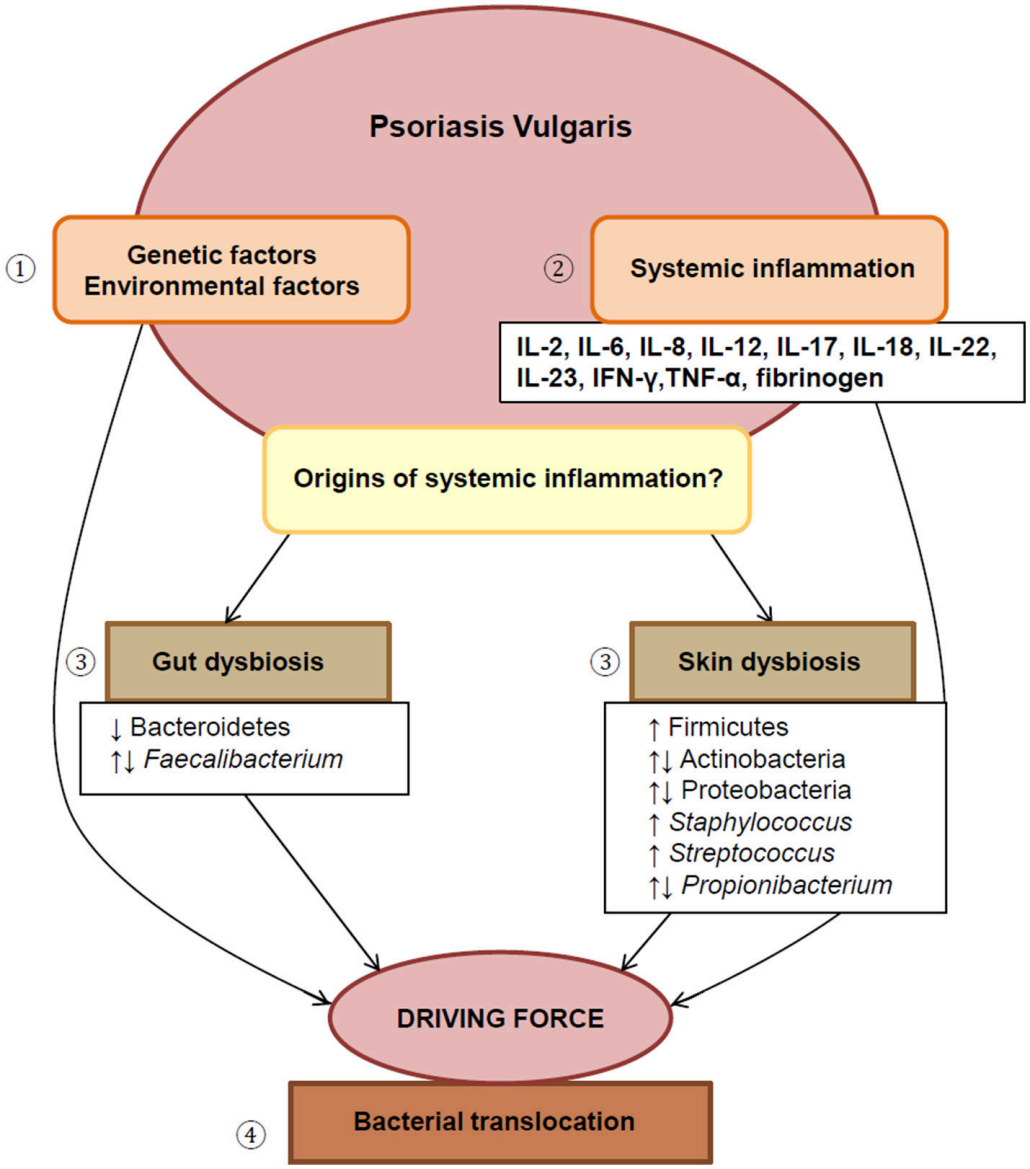
An overview of this paper. (1) The etiology of PV involves complex interplay between genetic and environmental factors. (2) This disease displays localized as well as systemic inflammation, reflected by the presence of various dysregulated inflammatory markers. (3) Dysbiosis of both the gut and skin microbiome are suggested as possible drivers of chronic systemic inflammation, (4) by facilitating the translocation of bacteria from these sites into systemic circulation. IL, interleukin; IFN-γ, interferon-gamma; TNF-α, tumor necrosis factor-alpha.

Psoriasis is a non-communicable, chronic inflammatory skin condition, which causes the rapid proliferation of skin cells, forming plaques of thickened skin covered in scales. According to the global report on psoriasis by the World Health Organization, this disease affects approximately 100 million individuals worldwide (WHO, 2016). We have argued previously (reviewed in Kell and Pretorius, 2018), that most of these chronic inflammatory diseases share significant elements in their etiologies. Thus various comorbidities are associated with this condition (Nestle et al., 2009; Boehncke and Schön, 2015; Kell and Pretorius, 2015b; Brooks, 2018). Psoriasis considerably impairs patients' quality of life and may also contribute to psychological stress in a multitude of these patients (Langley et al., 2005; Nestle et al., 2009). Psoriasis vulgaris (PV), or chronic plaque psoriasis, is the most common clinical variant and is the focus of this paper, where we will review

literature that delineates the systemic inflammatory nature of this condition. Specifically, we aim to emphasize changes in the gut and skin microbiome associated with PV. We will also discuss the potential of dysregulated circulating inflammatory markers with a bacterial origin as a driving force of the chronic systemic inflammation observed in PV. See Figure 1 for an overview (and Supplementary Material for notes on the healthy (normal) microbiome).

## The Systemic Inflammatory Nature of PV

The etiology of PV is currently not well-understood and involves a complex interplay between an individual's genetic predisposition and environmental factors (Gao et al., 2008; Nestle et al., 2009; Egeberg et al., 2016). For several years, PV has been viewed as a condition that is merely “skin-deep,” and thus inflammation was thought to be limited to the skin. However, this has been proved untrue in recent years, as new research also implicates a more general peripheral inflammation in PV.

The basic pathogenesis of PV involves aberrant cross-talk between the innate and adaptive immune systems, with central roles for the interleukin-23/interleukin-17 (IL-23/IL-17) axis, tumor necrosis factor-alpha (TNF-α), and particular skin cells such as dendritic cells and keratinocytes (Boehncke and Schön, 2015). Briefly, in response to a stressor, such as physical trauma, dendritic cells in the skin are activated and present an unknown antigen to naïve T lymphocytes (Ogawa et al., 2018). Subsequently, activated T lymphocytes differentiate into type 1 T helper (Th1), type 17 T helper (Th17), and type 22 T helper (Th22) cells. Dendritic cells, along with macrophages, B and T lymphocytes, secrete IL-23 which stimulates Th17 cells to differentiate and proliferate (Ogawa et al., 2018). Th17 cells secrete IL-17A, which in turn stimulates keratinocytes to produce various cytokines and chemokines (Woo et al., 2017; Ogawa et al., 2018). In addition, IL-23 stimulates the secretion of IL-22 from Th17 cells, which promotes the hyperproliferation of keratinocytes (Boehncke and Schön, 2015; Ogawa et al., 2018). Dendritic cells and Th17 cells also secrete TNF-α, which activates the nuclear factor-kappa B (NF-κB) signaling pathway (Nelson, 2004; Ashall et al., 2009; Boehncke and Schön, 2015; Ogawa et al., 2018). These dysregulated inflammatory molecules necessarily circulate through the cardiovascular system, accounting for the systemic inflammatory profile of these individuals. Other dysregulated markers may include, C-reactive protein, fibrinogen, interferon (IFN)-α, IFN-γ, IL-2, IL-6, IL-8, IL-12, IL-15, and IL-18 (Arican et al., 2005; Rashmi et al., 2009; Bai et al., 2018). These inflammatory molecules may contribute to the development of comorbid diseases. TNF-α specifically, is implicated in the pathogenesis of both PV and atherosclerosis. It has been found that TNF inhibitors have positive effects on cardiovascular biomarkers (Famenini et al., 2014). PV may also promote the development of type II diabetes mellitus (T2DM), due to the complex interactions between adipokines and pro-inflammatory cytokines associated with PV (Fitzgerald et al., 2014). Particularly, IL-6, IL-8, IL-17, IL-18, and TNF-α may contribute to the development of insulin resistance (Fitzgerald et al., 2014). Moreover, TNF-α inhibitors were positively associated with insulin sensitivity in psoriatic individuals with T2DM (Al-Mutairi and Shabaan, 2016). A link also exists between depression and PV. Elevated levels of cytokines may influence the development of depression and IL-6 appears to play a prominent role (Koo et al., 2017).

## The Driving Force of Systemic Inflammation in PV: an Aberrant Gut/Skin Microbiome?

The human microbiome displays a high degree of variation at inter- and intrapersonal level (Costello et al., 2009). Consequently, it has been impossible to define a “healthy” microbiome (Dave et al., 2012). However, it has become apparent that certain compositions of microbes may promote both health and disease (Blum, 2017). The term “dysbiosis” refers to alterations in the composition of the microbiota and has been implicated in the etiology of various diseases (de Oliveira et al., 2017; Kell and Pretorius, 2018; Kho and Lal, 2018).

Dysbiosis of both the gut and skin microbiome has been associated with psoriasis (Gao et al., 2008; Cohen et al., 2009; Fahlén et al., 2012; Alekseyenko et al., 2013; Li et al., 2013; Scher et al., 2015; Egeberg et al., 2016; Eppinga et al., 2016; Tett et al., 2017; Codoñer et al., 2018). Additionally, dysbiosis of the gut microbiota may contribute to the development of a leaky gut, facilitating bacterial translocation, which may act as a driving force of the inflammatory response (Mu et al., 2017). This is due to the shedding of functionally significant inflammagens, such as lipopolysaccharide (LPS) and lipoteichoic acid (LTA), by Gram-negative and Gram-positive bacteria, respectively (Kell and Pretorius, 2018). Furthermore, evidence is accumulating that suggests a link between the gut and skin, namely the gut-skin axis. It may be possible that dysbiosis of the gut microbiome may alter systemic immunity, resulting in dyshomeostasis and impaired functioning of the skin (Vaughn et al., 2017; Salem et al., 2018).

## Gut Dysbiosis

It is well-established that gut dysbiosis co-presents with psoriasis (Cohen et al., 2009; Li et al., 2013; Scher et al., 2015; Egeberg et al., 2016; Eppinga et al., 2016; Codoñer et al., 2018). There is also a significant association between psoriasis and inflammatory bowel disease (IBD) and immune-related clinical conditions including those affecting the gastrointestinal tract; and in fact, treatments for psoriasis are also used for IBD (Pietrzak et al., 2017; Whitlock et al., 2018).

However, very few studies have characterized alterations in the composition of the gut microbiome of psoriatic individuals. Scher et al. (2015) evaluated the relative changes in the gut microbiome of patients with psoriasis of the skin (PS) as well as patients with psoriatic arthritis (PA), compared to control subjects. A significant decrease in microbial diversity was observed in PS and PA patients (*p* < 0.05) (Scher et al., 2015). Indeed, a loss of gut microbial diversity is associated with an array of human diseases (Mosca et al., 2016). In PS patients, the relative abundance of the Actinobacteria and Bacteroidetes phylyms were reduced (*p* < 0.05), while *Coprobacillus, Ruminococcus*, and *Parabacteroides* were decreased at the genus level (Scher et al., 2015). Similar results were obtained by Codoñer et al. (2018), who found *Bacteroides* (a genus belonging to Bacteroidetes) to be decreased. *Bacteroides* is known to play an immunomodulatory role in the gut through the production of polysaccharide A which activates regulatory T cells (Mosca et al., 2016). Therefore, a decrease in this genus may result in an altered immune response to gut microbiota, further contributing to dysbiosis. Codoñer et al. also revealed the existence of a core microbiome in PV, which differed from that of healthy individuals (Codoñer et al., 2018). The psoriatic microbiome was characterized by an increase in the abundance of *Faecalibacterium* and a decrease in *Bacteroides* spp. Furthermore, Eppinga et al. (2016) found that a similar dysbiosis occurs in the gut microbiome in both IBD and psoriasis patients. The study compared the abundance of *Faecalibacterium prausnitzii* and *Escherichia coli* in patients with psoriasis (including PV, guttate psoriasis and palmoplantaris pustulosis) and patients with both psoriasis and IBD (CD, Crohn's disease and UC, ulcerative colitis) to controls (Eppinga et al., 2016). The authors reported a decrease in the beneficial *Faecalibacterium praunitzii* in PV (*p* < 0.001) and IBD (*p* < 0.001). The abundance of *Faecalibacterium praunitzii* was reduced to a greater extent in patients with both IBD and psoriasis, when compared to patients with only one of the conditions. These species produce butyrate, which inhibits the NF-κB pathway, thereby inhibiting the inflammatory response (Hiippala et al., 2018). A significant increase was also observed in the relative abundance of *Escherichia coli* in psoriasis patients (*p* = 0.002), as well as in patients with concomitant IBD and psoriasis (*p* < 0.001).

IBD has been associated with psoriasis since the 1970s (Verbov, 1973; Yates et al., 1982; Lee et al., 1990). However, this association has largely been disregarded. Initial reports of the association between psoriasis and CD, as well as UC, were confirmed by Cohen et al. (2009). The study cohort comprised 12,502 patients and 24,285 control subjects (Cohen et al., 2009). The prevalence of CD (0.5%) was significantly increased in individuals diagnosed with psoriasis, when compared to controls (0.2%) (*p* < 0.001). Similarly, a higher prevalence of UC was observed in patients with psoriasis (0.5%) than the general population (0.3%) (*p* = 0.002). Li et al. (2013) aimed to ascertain the association between the incidence of CD and UC, and psoriasis in a US population of women. Data were obtained from 174,476 women. The authors reported that although the incident risk was increased for CD, this was not the case for UC (Li et al., 2013). Patients with psoriasis exhibited a greater risk for the development of CD with a relative risk of 3.86 (95% confidence interval). Furthermore, the risk of developing IBD after the onset of psoriasis was also evaluated by Egeberg et al. (2016) in a Danish population. The study included a total of 5,554,100 subjects. The authors reported a significant increase in the risk for CD in patients with mild psoriasis, while a 2- and 3-fold increase in the risk was observed in severe psoriasis and psoriatic arthritis, respectively (Egeberg et al., 2016). Comparable results were obtained when psoriasis and UC was considered. Although none of these studies was able to determine causality, due to being observational, it is apparent that an association does indeed exist between PV and IBD.

## Skin Dysbiosis

Dysbiosis of the skin microbiome in individuals diagnosed with psoriasis has become a recurrent theme. However, recent studies describing the relative changes in microbial communities are limited (Gao et al., 2008; Fahlén et al., 2012; Alekseyenko et al., 2013; Tett et al., 2017). In a seminal study by Gao et al. (2008) the composition of the skin microbiome in lesional skin of patients with psoriasis was compared to uninvolved skin of the same patient as well as control subjects. The authors reported that all skin types were characterized by the presence of three phyla, namely Actinobacteria, Firmicutes, and Proteobacteria. Similar findings have been reported by other studies (Fahlén et al., 2012; Alekseyenko et al., 2013). Gao et al. (2008) also reported greater diversity in lesional skin than in uninvolved or healthy skin. In contrast, three other studies observed a trend toward decreased diversity in lesional skin (Fahlén et al., 2012; Alekseyenko et al., 2013). This latter observation is in accordance with literature, as it has been suggested that decreased diversity of the skin microbiome is associated with unhealthy skin (Wallen-Russell and Wallen-Russell, 2017; Dréno et al., 2018).

At the phylum level, an increase in the relative abundance of Firmicutes has been found in psoriatic skin (Gao et al., 2008; Alekseyenko et al., 2013). However, discrepant results have been obtained with regards to the abundance of Actinobacteria and Proteobacteria. Gao et al. (2008) observed a decrease in Proteobacteria (*p* < 0.001) and Actinobacteria (*p* < 0.01) in lesional skin. However, another study found that Proteobacteria were more abundant in lesional skin of the trunk (*p* = 0.011), while Actinobacteria were underrepresented in affected skin compared to healthy skin (*p* = 0.034) (Fahlén et al., 2012). Additionally, Alekseyenko et al. (2013) identified two cutaneotypes in their study cohort, where lesional skin was associated with the cutaneotype characterized by a higher abundance of Actinobacteria and Firmicutes (*p* < 0.01). At the genus level, *Streptococcus* (*p* < 0.001) and *Staphylococcus* have been found to be more abundant in lesional skin by Gao et al. Additionally, Fahlén and co-workers observed that the abundance of *Streptococcus* spp. were increased in affected skin, while *Staphylococcus* were detected more frequently in the skin of controls (Fahlén et al., 2012). In contrast, Tett et al. found *Staphylococcus* to be significantly increased in lesional skin (*p* = 0.043). A role for particularly *Staphylococcus aureus* and *Streptococcus pyogenes* has been proposed in the pathogenesis of PV (Tomi et al., 2005; Weisenseel and Prinz, 2005; Fry and Baker, 2007). Moreover, Alekseyenko et al. (2013) observed that the combined relative abundance of the Gram-positive *Corynebacterium, Propionibacterium, Staphylococcus*, and *Streptococcus* was increased in lesional skin. In contrast, Gao et al. (2008) found a progressive underrepresentation of *Propionibacterium* (*p* < 0.001) in affected skin compared to the skin of controls, followed by unaffected and control skin of individuals diagnosed with psoriasis. *Propionibacterium* has been established as a protective commensal bacteria, as this genus produces anti-microbial substances and possesses immunomodulatory properties (Christensen and Brüggemann, 2014). Therefore, the conflicting results may reflect a disrupted ecology of the microbiota in psoriasis (underrepresentation), while an increase may initially be protective (overrepresentation). “Next generation” whole-genome shotgun-metagenomic sequencing was utilized for the first time by Tett et al. (2017) to study microbial communities, as well as changes in the functionality of these communities in lesional and unaffected skin from PV patients. The authors emphasized complexities when evaluating changes in the skin microbiome, as affected skin from different skin sites exhibited divergent trends, for example at species level, diversity decreased in lesional skin from the ear (*p* = 0.008), while affected skin from the elbow displayed greater diversity.

## Could Bacterial Translocation via the Gut or Skin Play a Role in PV? A Hypothesis

Blood is commonly regarded as a sterile environment as actively growing and replicating microbes are considered to be absent (Damgaard et al., 2015; Kell and Pretorius, 2015a; Potgieter et al., 2015; Gosiewski et al., 2017). However, dormant or non-replicating bacteria may be present in the systemic circulation (Seubert et al., 2001; Urban et al., 2006; Thwaites and Gant, 2011; Potgieter et al., 2015; Kell and Pretorius, 2018). These bacteria do not form colonies when plated directly, have sometimes been referred to as “unculturable,” and might therefore also be regarded as non-viable, as classical microbiology equates viability with culturability (Postgate, 1969; Kell et al., 1998). However, bacteria in the blood are rather in a physiologically dormant state, considered to be neither “dead” nor “alive,” since by definition, dormant organisms can be resuscitated, in certain cases by particular molecules (Kaprelyants et al., 1993; Kell and Pretorius, 2015a).

Dysbiosis may promote the translocation of microorganisms from the gut into the bloodstream in many conditions (Bischoff et al., 2014; Fukui, 2016; Kell and Pretorius, 2018). Translocation from the gut may occur via three entry points, namely dendritic cells, a compromised intestinal epithelial barrier, and M cells (Potgieter et al., 2015). As mentioned earlier, these translocating microbes may not be metabolically active or replicating, yet they may contribute to maintaining a chronic, low-grade inflammatory state in the host. Periodically, dormant bacteria may shed their cell wall components, such as LPS and LTA, especially when awakened (Kell and Pretorius, 2015a; Potgieter et al., 2015). Both of these inflammagens will induce the production of inflammatory cytokines and chemokines, activating the innate and adaptive immune systems (Kell and Pretorius, 2015a, 2018; Potgieter et al., 2015). Our literature searches strongly suggest a bacterial involvement in PV (see Table 1).

**Table 1:** A summary of the most prominent findings that imply a bacterial component in PV.

**Study design**	**Findings**	**References**
Administration of benzathine penicillin in PV patients (*n* = 30) for 2 years	Significant improvement at 12 weeks (*p* < 0.01) 91–100% improvement at 2 years (*p* < 0.01) *Streptococcus* suggested as initial trigger of guttate psoriasis, with PV as delayed manifestation	Saxena and Dogra, 2005
Evaluating levels of *Streptococcus pyogenes* immunoglobulin G (IgG)-reactive proteins in PV patients (*n* = 8) compared to controls	IgG-reactive proteins were significantly increased in patients (*p* = 0.0009)	El-Rachkidy et al., 2007
Detecting levels of genes coding for Streptococcal and Staphylococcal super-antigens in psoriasis patients (*n* = 22)	Super-antigens were detected in 59% of patients	El Ferezli et al., 2008
Evaluating the effect of Streptococcal DNA on peripheral blood mononuclear cells (PBMCs) isolated from PV patients	Enhanced proliferation of PBMC in patients (*p* = 0.032)	Cai et al., 2009
Administration of azithromycin in PV patients (*n* = 30) for 48 weeks	Significant improvement at 12 weeks 91–100% improvement in 18 patients and 60–90% improvement in six patients at 48 weeks six patients experienced a relapse after 1 year	Saxena and Dogra, 2010
Determining frequency of subclinical microbial infection in PV patients (*n* = 195) compared to controls	Infection with *Staphylococcus* and *Streptococcus* were detected in 68% of patients, compared to 11% in controls	Bartenjev and Potocnik, 2000

As discussed above, dysbiosis of both the skin and gut microbiome is found in association with psoriasis. Therefore, we hypothesize that dysbiosis of the microbiota in individuals with PV facilitates the translocation of bacteria from the gut and skin into the bloodstream, which may act as a driving force of the chronic, systemic inflammation in these individuals (Zákostelská et al., 2016). There is some evidence that supports bacterial translocation in the context of PV. Bacterial DNA has been detected in monocytes isolated from patients with PV (Okubo et al., 2002). The authors reported that bacterial DNA was significantly increased in PV patients, compared to healthy controls. Munz et al. (2010) also found bacterial DNA in peripheral blood of patients with guttate psoriasis as well as PV. *Staphylococcus* spp. were mainly identified in patients with PV, while *Streptococcus* spp. were found in association with guttate psoriasis (Munz et al., 2010). This last study is of particular interest, as the relative abundance of these genera is increased in lesional skin of psoriatic patients (Gao et al., 2008; Fahlén et al., 2012; Alekseyenko et al., 2013). Moreover, these observations might suggest the possible translocation of bacteria from the skin into the bloodstream. Bacterial DNA has also been detected by Ramírez-Boscá et al. in peripheral blood of patients (*p* < 0.05) in their study cohort, with (perhaps surprisingly) *Escherichia coli* identified as the main source. Interestingly, bacterial DNA was not found in patients with guttate and inverse psoriasis. The authors also reported that cytokine levels correlated significantly with the presence of bacterial DNA (*p* < 0.001). This study proposed the gut as possible origin of these bacteria as most of the detected species were associated with the intestinal microbiome (Ramírez-Boscá et al., 2015). A recent study also suggests that the presence of *Prevotella* spp. in conjunction with an increase in the ratio of *Faecalibacterium:Bacteroides* in the gut of PV patients, may facilitate bacterial translocation (Codoñer et al., 2018).

From these studies it is evident that bacterial translocation may take place in PV. However, more research is needed to determine if bacterial species in the circulation originate primarily from the gut, the skin or indeed the oral cavity. Another important aspect would be to determine what specific changes in the gut and skin microbiome or other external factors facilitate subsequent bacterial translocation. For instance obesity is well-known to relate to translocation, and there is significant association between obesity and psoriasis (Armstrong et al., 2012; Carrascosa et al., 2014; Jensen and Skov, 2017).

## Iron Dysregulation

Common to all chronic inflammatory diseases is iron dysregulation, that can resuscitate dormant microbes (Kell, 2009; Kell and Pretorius, 2018). Here too there is clear evidence for the involvement of a deranged iron metabolism (Trenam et al., 1992; Wojas-Pelc and Marcinkiewicz, 2007). One possible strategy is thus the use of iron chelators (Hider et al., 1992); see also Supplementary Material.

## Implications for Treatment

Recently it was also suggested that treatment of psoriasis might begin with altering the bowel flora toward normality, and therapy might include the use of appropriate antibiotics (Wang et al., 2009; Waterhouse et al., 2009; Youssef et al., 2011; Alzolibani and Zedan, 2012; Baros et al., 2014; Kadam et al., 2015; Zákostelská et al., 2016; Allen et al., 2017; Ely, 2018). This avenue should be investigated further. Also, the use of prebiotics might be a useful avenue to investigate (Ely, 2018). Importantly, an improved knowledge of the basis for psoriatic lesions is only of real value if it leads to beneficial therapies. Since our main point here is that a chief driver is the atopobiotic appearance of unwanted microbes in these skin lesions, an obvious suggestion is the use of those antibiotics that can penetrate into host cells via membrane transporters (Kell et al., 2013; Kell and Oliver, 2014; Prideaux et al., 2015). This suggestion seems to be borne out in practice (Saxena and Dogra, 2005, 2010; Walecka et al., 2009). Finally, if inflammation is a cause and not a manifestation, one may anticipate that anti-inflammatories might also be of value (Aggarwal and Harikumar, 2008; Kang et al., 2016; Chen et al., 2017; Sharma et al., 2017; Kerley et al., 2018; Owczarczyk-Saczonek et al., 2018).

## Conclusion

“Coherence” describes the idea that if several independent lines of evidence lead to a similar conclusion, that conclusion is thereby strengthened (Thagard, 1999, 2008). Here we have brought together evidence in order to formulate such a coherence in our scientific understanding of PV. Specifically, our aim was to bring together multiple lines of evidence suggesting the role of a leaky gut, the presence of inflammation and aberrant microbiome, both in the gut and the skin, and how this may be directly linked to the development of PV. PV is a chronic inflammatory condition characterized by the hyperproliferation of keratinocytes and infiltration by immune cells. Aside from inflammation localized to the skin, this disease also displays systemic inflammation reflected in the multitude of dysregulated inflammatory markers in the blood of these individuals. Consequently, there are a great many comorbidities and other epidemiological data that give vital clues to their etiologies. An aberrant skin microbiome has been associated with psoriasis, with specific alterations in the relative abundance of Firmicutes, Actinobacteria and Proteobacterium. Additionally, *Streptococcus* and *Staphyloccocus* have been found to be increased in affected skin. Gut dysbiosis, characterized by a decrease in microbes with anti-inflammatory and immunomodulatory properties, also seems to be prevalent in individuals diagnosed with PV. Moreover, an association between IBD and psoriasis is evident, with both conditions exhibiting a similar dysbiosis. We suggest that dysbiosis of the skin and gut microbiota may contribute to PV by facilitating the translocation of microorganisms into the blood, and that it is indeed worth considering microbes as etiological agents in this disease.

A question that needs to be further investigated is whether the changes in gut bacteria in psoriasis patients are a cause or a result (or both), and how their presence may be managed in a treatment regime. In particular, microbes may appear where they should not be present, and may shed inflammagens, such as LPS and LTA, both of which will result in an inflammatory responses. Therefore, detailed characterization of the microbiome in PV, and the use of suitable pro- and anti-biotics may have important implications in terms of preventing, diagnosing and treating this disease.

## Author Contributions

MV wrote the paper. EP study leader, co-wrote paper, and corresponding author. DK edited the paper and co-corresponding author. All authors reviewed the manuscript.

### Conflict of Interest Statement

The authors declare that the research was conducted in the absence of any commercial or financial relationships that could be construed as a potential conflict of interest.
