# Supplementary material for: Bacterial Dysbiosis and Translocation in Psoriasis Vulgaris
Source: Front Cell Infect Microbiol. 2019 Feb 4;9:7. doi: 10.3389/fcimb.2019.00007 (PMC6369634; doi:10.3389/fcimb.2019.00007)
Supplement: Supplementary file 1 [file Data_Sheet_1.pdf]

Supplementary material

The normal microbiome

Conventionally, culture-based methods were primarily used to describe the human microbiome, although nearly 80% of human-associated microbes are as yet uncultured (Lagier et al., 2015; Peterson et al., 2009). Identification of such uncultured microorganisms without the need for their controlled replication became possible with the advent of modern high-throughput technologies, such as 16S rRNA sequencing (Jo et al., 2015; Lagier et al., 2015). This method relies on the more or less conserved and necessarily present prokaryotic 16S ribosomal gene to classify microorganisms (Jo et al., 2015). Upon completion of the sequencing of the human genome, the Human Microbiome Project (HMP) was launched to elucidate the interactions between humans and microbes. The HMP aimed to characterise human-associated microbial communities to such an extent, that it would be possible to determine microbial influence on and association with human health and disease (Peterson et al., 2009). Samples across 18 body sites of more than 250 volunteers were collected and sequenced with the goal to define a core microbiome at each specific site (Peterson et al., 2009). The HMP and related projects have given researchers new insights into the human microbiota, revolutionising research in this field.

The human gastrointestinal microbiome contains approximately 10-100 trillion microbes and its content of genes may outnumber human genes by 100-fold (Zhu et al., 2010). Microbial diversity is determined by a range of factors, which include diet, health status of the host, genetic variation, mode of delivery (vaginal or caesarean section), geographical location, and the use of pharmaceutical drugs (Blum, 2017; Maier et al., 2018; Singh et al., 2017; Turnbaugh et al., 2009; Zhang et al., 2018; Zhu et al., 2010). Moreover, age, gender and ethnicity may also contribute to a variability in composition of the microbiota (Dave et al., 2012). Several crucial functions are fulfilled by commensal microbes, such as the breakdown of otherwise indigestible oligosaccharides, the synthesis of essential amino acids and vitamins, the metabolism and/or removal of xenobiotics, protection against invading pathogens, and conserving the integrity of the gastrointestinal epithelium (Dave et al., 2012; Kurokawa et al., 2007; Salem et al., 2018; Zhu et al., 2010). Moreover, microbial communities may have close interactions with the gastrointestinal immune system of the host, such as the gut associated lymphoid tissues (Zhu et al., 2010). Commensal microbes and their cell wall components are recognised by pattern recognition receptors, such as Toll-like receptors and NOD-like receptors, and prime the innate immune response (Kell and Pretorius, 2015; Kho and Lal, 2018; Kosiewicz et al., 2014; Salem et al., 2018). With regard to the adaptive immune system, the gut microbiome is responsible for maintaining a balance between the differentiation of effector and regulatory T cells, as well as the secretion of immunoglobulin A (de Oliveira et al., 2017; Salem et al., 2018). Composition of the gut microbiota differs considerably from one individual to another (Byrd et al., 2018; Grice et al., 2009), even so the adult microbiome has been characterised by the presence of two main phyla, namely Bacteroidetes and Firmicutes (Donaldson et al., 2015; Eckburg et al., 2005; Hiippala et al., 2018).

Normal human skin houses a myriad of microorganisms and may contain as many as two million bacteria per square centimetre of skin (Wong et al., 2013). In addition to the presence of bacteria, the skin is also colonised by fungi, viruses and arthropods (Wang and Jin, 2018). However, we will be focussing mainly on bacteria here. Microorganisms comprising the skin microbiota can be categorised into two groups, namely residential and transient microbes (Dréno et al., 2016). Residential microbes that are omnipresent on the skin are considered to be commensal, while transient microbes are present for only brief periods of time. Various factors may influence

microbial diversity, such as age, gender, the use of antibiotics, genetic variation and hygiene (Dréno et al., 2016; Prescott et al., 2017). The skin microbiome serves as a barrier against invading pathogens, it modulates the immune response, and it aids in the breakdown of natural products (Byrd et al., 2018). Human skin has several distinct anatomical niches, termed skin sites, each presenting with unique physiological characteristics. These sites can be categorised as dry, moist or sebaceous (Byrd et al., 2018). Dry skin sites are the most diverse and primarily colonised by *Propionibacterium*, *Corynebacterium* and *Streptococcus* spp (Byrd et al., 2018). Moist skin sites, such as the axillae and antecubital fossa (inner elbow), are characterised by the presence of *Corynebacterium*, *Staphylococcus* and *Propionibacterium* (Byrd et al., 2018; Grice et al., 2009). Sebaceous skin sites are typically colonised by *Propionibacterium*, *Staphylococcus* and *Corynebacterium* and include areas such as the alar crease and the back (Byrd et al., 2018; Grice et al., 2009). Moreover, sweat and sebaceous glands as well as hair follicles, will harbour unique microbial communities (Grice and Segre, 2011). Microorganisms may have interactions with these unique microenvironments. The formation of body odour is attributed to bacterial processing of secretions by apocrine glands (Troccaz et al., 2015) and *Propionibacterium acnes* hydrolyses free fatty acids from sebum, which contributes to maintaining the acidic pH of the skin (Grice and Segre, 2011).

A role for iron dysregulation?

One part of the Iron Dysregulation and Dormant Microbes (IDDM) hypothesis is that the normally non-replicating bacteria involved are limited in the availability to them of free iron (Kell and Pretorius, 2018). This implies that such chronic inflammatory diseases should be accompanied by iron dysregulation, and this is indeed the case (Kell, 2009; Kell and Pretorius, 2014; Ponikowska et al., 2015; Rashmi et al., 2012; Trenam et al., 1992a, 1992b; Wojas-Pelc and Marcinkiewicz, 2007). Flo and co-workers (2004) demonstrated that lipocalin-2 (LCN-2) levels increased in response to a bacterial siderophore. Invading bacteria synthesise siderophores which are small molecules that sequester iron from the host (or environment) and transport it into the microbe. LCN-2 was considered to have exerted its effect by binding to the siderophore, thereby limiting the availability of iron to bacteria (Flo et al., 2004). In PV specifically, it has been observed that serum LCN-2 is significantly elevated in patients with this condition compared to that in control subjects ($p < 0.05$) (Kamata et al., 2012). This suggests the possible dysregulation of iron metabolism in response to bacterial infection. Additionally, the authors also reported that LCN-2 correlated significantly with the levels of IL-6 ($p < 0.05$) and TNF-α ($p < 0.01$).

References

Blum, H. E. (2017). The human microbiome. *Adv. Med. Sci.* 62, 414–420. doi:10.1016/j.advms.2017.04.005.

Byrd, A. L., Belkaid, Y., and Segre, J. A. (2018). The human skin microbiome. *Nat. Rev. Microbiol.* 16, 143–155. doi:10.1038/nrmicro.2017.157.

Dave, M., Higgins, P. D., Middha, S., and Rioux, K. P. (2012). The human gut microbiome: Current knowledge, challenges, and future directions. *Transl. Res.* 160, 246–257. doi:10.1016/j.trsl.2012.05.003.

de Oliveira, G. L. V., Leite, A. Z., Higuchi, B. S., Gonzaga, M. I., and Mariano, V. S. (2017). Intestinal dysbiosis and probiotic applications in autoimmune diseases. *Immunology* 152, 1–12. doi:10.1111/imm.12765.

Donaldson, G. P., Lee, S. M., and Mazmanian, S. K. (2015). Gut biogeography of the bacterial microbiota. *Nat. Rev. Microbiol.* 14, 20–32. doi:10.1038/nrmicro3552.

Dréno, B., Araviiskaia, E., Berardesca, E., Gontijo, G., Sanchez Viera, M., Xiang, L. F., et al. (2016). Microbiome in healthy skin, update for dermatologists. *J. Eur. Acad. Dermatology Venereol.* 30, 2038–2047. doi:10.1111/jdv.13965.

Eckburg, P. B., Bik, E. M., Bernstein, C. N., Purdom, E., Dethlefsen, L., Sargent, M., et al. (2005). Microbiology: Diversity of the human intestinal microbial flora. *Science (80-.).* 308, 1635–1638. doi:10.1126/science.1110591.

Flo, T. H. T., Smith, K. D. K., Sato, S., Rodriguez, D. J. D. J., Holmes, M. A., Strong, R. K., et al. (2004). Lipocalin 2 mediates an innate immune response to bacterial infection by sequestrating iron. *Nature* 432, 917–921. doi:10.1038/nature03021.Published.

Grice, E. A., Kong, H. H., Conlan, S., Deming, C. B., Davis, J., Young, A. C., et al. (2009). Topographical and Temporal Diversity of the Human Skin Microbiome. *Science (80-.).* 324, 1190–1192. doi:10.1126/science.1171700.

Grice, E. A., and Segre, J. A. (2011). The skin microbiome. *Nat. Rev. Microbiol.* 9, 244–253. doi:10.1038/nrmicro2537.

Hiippala, K., Jouhten, H., Ronkainen, A., Hartikainen, A., Kainulainen, V., Jalanka, J., et al. (2018). The potential of gut commensals in reinforcing intestinal barrier function and alleviating inflammation. *Nutrients* 10. doi:10.3390/nu10080988.

Jo, J. H., Kennedy, E. A., and Kong, H. H. (2015). Research Techniques Made Simple: Bacterial 16S Ribosomal RNA Gene Sequencing in Cutaneous Research. *J. Invest. Dermatol.* 136, e23–e27. doi:10.1016/j.jid.2016.01.005.

Kamata, M., Tada, Y., Tatsuta, A., Kawashima, T., Shibata, S., Mitsui, H., et al. (2012). Serum lipocalin-2 levels are increased in patients with psoriasis. *Clin. Exp. Dermatol.* 37, 296–299. doi:10.1111/j.1365-2230.2011.04265.x.

Kell, D. B. (2009). Iron behaving badly: inappropriate iron chelation as a major contributor to the aetiology of vascular and other progressive inflammatory and degenerative diseases. *BMC Med. Genomics* 79, 1–79. doi:10.1186/1755-8794-2-2.

Kell, D. B., and Pretorius, E. (2014). Serum ferritin is an important inflammatory disease marker, as it is mainly a leakage product from damaged cells. *Metallomics* 6, 748–773. doi:10.1039/c3mt00347g.

Kell, D. B., and Pretorius, E. (2015). On the translocation of bacteria and their lipopolysaccharides between blood and peripheral locations in chronic, inflammatory diseases: the central roles of LPS and LPS-induced cell death. *Integr. Biol. (United Kingdom)* 7, 1339–1377. doi:10.1039/c5ib00158g.

Kell, D. B., and Pretorius, E. (2018). No effects without causes: the Iron Dysregulation and Dormant Microbes hypothesis for chronic, inflammatory diseases. *Biol. Rev.* 93, 1518–1557. doi:10.1111/brv.12407.

Kho, Z. Y., and Lal, S. K. (2018). The Human Gut Microbiome – A Potential Controller of Wellness and Disease. *Front. Microbiol.* 9, 1–23. doi:10.3389/fmicb.2018.01835.

Kosiewicz, M. M., Dryden, G. W., Chhabra, A., and Alard, P. (2014). Relationship between gut microbiota and development of T cell associated disease. *FEBS Lett.* 588, 4195–4206. doi:10.1016/j.febslet.2014.03.019.

Kurokawa, K., Itoh, T., Kuwahara, T., Oshima, K., Toh, H., Toyoda, A., et al. (2007). Comparative metagenomics revealed commonly enriched gene sets in human gut microbiomes. *DNA Res.* 14, 169–181. doi:10.1093/dnares/dsm018.

Lagier, J. C., Hugon, P., Khelaifia, S., Fournier, P. E., La Scola, B., and Raoult, D. (2015). The rebirth of culture in microbiology through the example of culturomics to study human gut microbiota. *Clin. Microbiol. Rev.* 28, 237–264. doi:10.1128/CMR.00014-14.

Maier, L., Pruteanu, M., Kuhn, M., Zeller, G., Telzerow, A., Anderson, E. E., et al. (2018). Extensive impact of non-antibiotic drugs on human gut bacteria. *Nature* 555, 623–628. doi:10.1038/nature25979.

Peterson, J., Garges, S., Giovanni, M., McInnes, P., Wang, L., Schloss, J. A., et al. (2009). The NIH Human Microbiome Project. *Genome Res.* 19, 2317–2323. doi:10.1101/gr.096651.109.

Ponikowska, M., Tupikowska, M., Kasztura, M., Jankowska, E. A., and Szepietowski, J. C. (2015). Deranged iron status in psoriasis: The impact of low body mass. *J. Cachexia. Sarcopenia Muscle* 6, 358–364. doi:10.1002/jcsm.12061.

Prescott, S. L., Larcombe, D. L., Logan, A. C., West, C., Burks, W., Caraballo, L., et al. (2017). The skin microbiome: Impact of modern environments on skin ecology, barrier integrity, and systemic immune programming. *World Allergy Organ. J.* 10, 1–16. doi:10.1186/s40413-017-0160-5.

Rashmi, R., Yuti, A. M., and Basavaraj, K. H. (2012). Enhanced ferritin/iron ratio in psoriasis. *Indian J. Med. Res.* 135, 662–665. Available at: https://www.scopus.com/inward/record.uri?eid=2-s2.0-84863228486&partnerID=40&md5=6a1a2a8f4cbf53e0d0575fdb08ee438c.

Salem, I., Ramser, A., Isham, N., and Ghannoum, M. A. (2018). The gut microbiome as a major regulator of the gut-skin axis. *Front. Microbiol.* 9, 1–14. doi:10.3389/fmicb.2018.01459.

Singh, R. K., Chang, H. W., Yan, D., Lee, K. M., Ucmak, D., Wong, K., et al. (2017). Influence of diet on the gut microbiome and implications for human health. *J. Transl. Med.*, 1–17. doi:10.1186/s12967-017-1175-y.

Trenam, C. W., Blake, D. R., and Morris, C. J. (1992a). Skin Inflammation: Reactive Oxygen Species and the Role of Iron. *J. Invest. Dermatol.* 99, 675–682.

Trenam, C. W., Dabbagh, A. J., Blake, D. R., and Morris, C. J. (1992b). The role of iron in an acute model of skin inflammation induced by reactive oxygen species (ROS). *Br. J. Dermatol.* 126, 250–256. doi:10.1111/j.1365-2133.1992.tb00654.x.

Troccaz, M., Gaïa, N., Beccucci, S., Schrenzel, J., Cayeux, I., Starkenmann, C., et al. (2015). Mapping axillary microbiota responsible for body odours using a culture-independent approach. *Microbiome* 3, 3. doi:10.1186/s40168-014-0064-3.

Turnbaugh, P. J., Ridaura, V. K., Faith, J. J., Rey, F. E., Knight, R., and Gordon, J. I. (2009). The Effect of Diet on the Human Gut Microbiome : A Metagenomic Analysis in Humanized Gnotobiotic Mice. *Sci. Transl. Med.* 1.

Wang, W. M., and Jin, H. Z. (2018). Skin Microbiome: An Actor in the Pathogenesis of Psoriasis. *Chin. Med. J. (Engl).* 131, 95–98. doi:10.4103/0366-6999.221269.

Wojas-Pelc, A., and Marcinkiewicz, J. (2007). What is a role of haeme oxygenase- 1 in psoriasis? Current concepts of pathogenesis. *Int. J. Exp. Pathol.* 88, 95–102.

Wong, V. W., Martindale, R. G., Longaker, M. T., and Gurtner, G. C. (2013). From germ theory to germ therapy: Skin microbiota, chronic wounds, and probiotics. *Plast. Reconstr. Surg.* 132, 854–861. doi:10.1097/PRS.0b013e3182a3c11e.

Zhang, N., Zhongjie, J., and Zuo, T. (2018). Time for food : The impact of diet on gut microbiota and human health. *Nutrition* 51–52, 80–85. doi:10.1016/j.nut.2017.12.005.

Zhu, B., Wang, X., and Li, L. (2010). Human gut microbiome: The second genome of human body. *Protein Cell* 1, 718–725. doi:10.1007/s13238-010-0093-z.
